# Reply to Mrs. Stopes' Letter

**Published:** 1920-11-20

**Authors:** 


### Reply to Mrs. Stopes' Letter.

[I regret that Mrs. Stopes forces me into a position of apparent antagonism, because I have a great respect for much of her work, and I am sure that, *tête-à-tête,* we should find much more to agree than to differ upon. However, I must take up her challenge.

On page 51, when discussing childbirth, she speaks of "agonising torments" and the "abiding horror" which "is so great that not a few women refuse to bear another child." Without denying for a moment that childbirth is a painful thing, I submit that this language is exaggerated and likely to produce wholly false impressions in the minds of those who read it; nay, I know of one case where it has already done so. It ignores entirely the vast diminution of maternal suffering brought about by anæsthetics, and will encourage neurotic women in the shirking of their responsibilities. In an experience now fairly large, I know of but one case in which the "abiding horror" clause stands good: and that is a woman with a contracted pelvis who had two children a few years ago in a remote village in Wales, where the doctor was a survival from a past generation and "did not believe in anæsthetics." I further contest the statement that "in earlier and simpler civilisations the women were probably better cared for and better fitted for motherhood than the majority of women are to-day." In fact, all this chapter is full of exaggerations, such as "the ratings and peevish fury of callous men," amongst whom I hope Mrs. Stopes does not include myself. Her argument that the ever-increasing size of the brain at birth (itself by no means scientifically established) will necessitate universal Cæsarean section takes no account of the equally probable increase in pelvic diameters amongst women, and also overlooks important surgical considerations, such as those discussed by Dr. Eardley Holland in a recent contribution to the *Lancet* on the subject of this operation.

The first sentence on page 57 is surely too absurd for Mrs. Stopes to maintain seriously: I hope, on reconsideration, she will admit this. Pages 59 and 60, too, contain palpable over-statements. On page 63 we find again an outburst of sexual antipathy against the "average gross and overbearing male," which is true neither to fact nor to the balance of opinion among women.

Then, in dealing with nausea in pregnancy, Mrs. Stopes starts by declaring that no expectant mother need experience it; and that it can be "entirely conquered." Later on she hedges, admitting that the measures she advocates will suffice "in all but the exceptional and predisposed." Admitting that her advice is good on the whole, this sentence is merely question-begging. Nor, in my view, is she correct when she maintains that trouble with varicose veins in pregnancy can be prevented by diet. Page 107 contains a statement about electricity which, one feels, must have escaped the notice of Mrs. Stopes' physiologist friend; and it may be doubted whether he concurs in her belief that an unborn child "is apparently to some extent conscious of the nearness of its father": there is an old saw which would make this a very wise child indeed.

Again, Mrs. Stopes declares that premature birth in the eighth month is less frequent than in the seventh—a quite unfounded statement. From the context, it seems probable that when Mrs. Stopes speaks of "in the eighth month" she really means "in the ninth (calendar) month," for she speaks of the popular belief that it is more difficult to rear a child born in the eighth month than in the seventh, "though this does not appear to be true." It certainly is utterly false, for to rear a child born earlier than seven months ("in the seventh month") from conception was practically unknown until quite recent times, and even now is highly unusual. But the popular belief she speaks of does not refer to children born "in" the eighth and seventh months respectively, but to those born "at" eight months or seven months—*i.e.,* in the ninth and eighth (calendar) months. In a later chapter I find the author aware that the normal duration of pregnancy is about ten lunar months. I am giving her the benefit of the doubt in this paragraph; or, if she was referring to lunar months, she is still more in error.

Yet again, I contest Mrs. Stopes' *ipse dixit,* unsupported as it is by any argument or any statistical basis, that children born to mothers under thirty-five may be "healthy, jolly little animals," but will have "no more brains than are sufficient to see them creditably through life"; whereas those parents who want to procreate "one of the *master* minds" (her italics) should arrange for the mother to have her first and only baby between the ages of thirty-five and forty-five. I still hold that "grotesque" is the right word to apply to statements of this sort.—The Reviewer.]

